# A qualitative assessment of the acceptability of human papillomavirus self‐sampling and informational materials among diverse populations

**DOI:** 10.1002/cam4.70033

**Published:** 2024-07-23

**Authors:** Amanda F. Petrik, Jennifer S. Rivelli, Alison J. Firemark, Cheryl A. Johnson, Blake W. Locher, Sara Gille, Matthew J. Najarian, Alexandra M. Varga, Jennifer L. Schneider, Beverly Green, Rachel L. Winer

**Affiliations:** ^1^ Kaiser Permanente Center for Health Research Portland Oregon USA; ^2^ Oregon Health and Sciences University/Portland State University School of Public Health Portland Oregon USA; ^3^ Kaiser Washington Health Research Institute Seattle Washington USA; ^4^ University of Washington School of Public Health Seattle Washington USA

**Keywords:** cervical cancer screening, diverse populations, HPV self‐test, qualitative interviews, self‐sampling

## Abstract

**Background:**

Disparities in cervical cancer screening rates among marginalized groups is a driver of inequalities in cervical cancer. Self‐sampling for human papillomavirus (HPV) testing is a newly emerging alternative to clinician‐performed testing to screen for cervical cancer, and has high potential to reduce screening barriers in under‐screened and marginalized groups. We study the acceptability in of HPV self‐sampling and informational materials among Black/African American, Hispanic/Spanish speaking, American Indian/Alaska Native and transgender/nonbinary populations.

**Methods:**

We conducted qualitative interviews with patients, ages 30–65, who were Black/African American, Hispanic, American Indian, and/or transgender/nonbinary individuals assigned female at birth. Telephone interviews were conducted in English or Spanish. Patients did not complete the test, rather were asked about the attractiveness, comprehensibility, and acceptability of the HPV self‐test, instructions, and messaging.

**Results:**

Among 23 completed interviews (5 American Indian/Alaska Native, 7 Hispanic [2 bilingual, 5 Spanish‐speaking], 5 Black/African American, and 6 transgender/nonbinary), patients from all groups thought the test was straightforward and convenient, and they would complete the test at home or in clinic. The transgender/nonbinary patients preferred at‐home testing. American Indian and transgender/nonbinary patients liked that the test might avoid pain, discomfort, and invasiveness. All patients liked the letter and instructions. All groups had specific suggestions for making the materials more culturally acceptable.

**Conclusions:**

The HPV self‐test and the instructions and materials for use were acceptable for a diverse group of patients. Tailored outreach and messaging should be considered to reduce screening disparities among groups that have been historically underserved by the medical system.

## 1 BACKGROUND

Differential use of cervical cancer screening across demographic groups has been found to create inequalities in diagnosis and survival rates of cervical cancer at later stages.^1^ Research has found cervical cancer screening is lower among non‐Hispanic Black patients and higher among Hispanic patients, compared to non‐Hispanic White patients,^2^ and that non‐Hispanic patients of races other than White were significantly less likely to receive a Pap test than White patients.^3^ Racial and ethnic variation in screening has caused differences in cervical cancer incidence; for every 100,000 newly diagnosed cases of cervical cancer, Black women attributed 8.9 new cases, Hispanic women 9.4, and White women 7.5 new cases.^4^, ^5^

Interventions to address disparities in cervical cancer screening have been a priority focus for American Indian/Alaska Native individuals; cervical cancer screening rates among American Indian/Alaska Native women (57.1%–65.0%) are well below the national average (73.5%).^6^ Population‐level health behaviors regarding cervical cancer screening have also varied among sexual gender minorities.^7^ Studies have found that gay and lesbian individuals have a decreased likelihood of following cervical cancer screening recommendations compared to heterosexual individuals.^8^, ^9^ Transgender men have a lower likelihood of accessing cervical cancer screening care than cisgender women.^10^ Those with intersecting sexual/gender and racial/ethnic identities that are associated with low screening rates are at particular risk of not receiving cervical cancer screening.^7^

Human papillomavirus (HPV) self‐sampling can address screening barriers given easy access and the flexible distribution options. HPV self‐sampling kits can be used in clinics as an alternative to a scheduled Pap and/or HPV test. It can also be distributed at other appointments (e.g., lab visits, or urgent care), or mailed to patients' homes. As fecal immunochemical testing proved for colorectal cancer screening, this type of screening frees up time and resources for clinical teams, and reduces costs.^11^ For patients, HPV self‐sampling can address provider time barriers to in‐clinic appointments and reluctance to screen based on negative experiences or attitudes towards pelvic exams. Self‐sampling methods will be important for populations that are resistant to screening, including victims of sexual assault or the transgender population.^12^ Thus, HPV self‐sampling may reduce screening barriers in under‐screened and marginalized groups, including rural residents, racial and ethnic minorities, sexual and gender minorities, individuals with a history of trauma or sexual abuse, and individuals with religious or cultural barriers to pelvic exams.

Despite widespread international adoption, only a few health systems in the US have adopted HPV self‐sampling. Once FDA approved, the shift to HPV self‐sampling will offer opportunity to reduce disparities among historically underserved populations. The trial conducted on directly mailing self‐sampling kits to patients in the US (*Self‐Testing options in the Era for Primary HPV Screening for Cervical Cancer* [STEP trial]) showed a 14% increase in HPV screening among those due or overdue in a well‐resourced healthcare system.^13^, ^14^ As this screening method is becoming more available, healthcare systems will be developing capacity to process HPV self‐tests.

To preemptively address disparities among populations that have been historically underserved, health systems and providers should understand acceptability of self‐sampling among specific underserved population groups and refine messaging to be able to effectively communicate about this new test with those patients. This paper reports the results of patient interviews on the acceptability of the HPV self‐sampling, as well as messaging and materials for this new method of cervical cancer screening, among Black/African American, Hispanic/Spanish‐speaking, American Indian/Alaska Native, and transgender/nonbinary populations.

## 2 METHODS

This study was conducted at Kaiser Permanente Northwest (KPNW), a large health system in Oregon and southwest Washington that provides care to more than 615,000 patients each year. 3.6% of KPNW patients are identified in the Electronic Health Record as Black, 9.9% as Hispanic, and 0.06% as American Indian/Alaska Native.^15^ Patients at KPNW are insured by either commercial insurance, Medicaid or Medicare.

**FIGURE 1:**
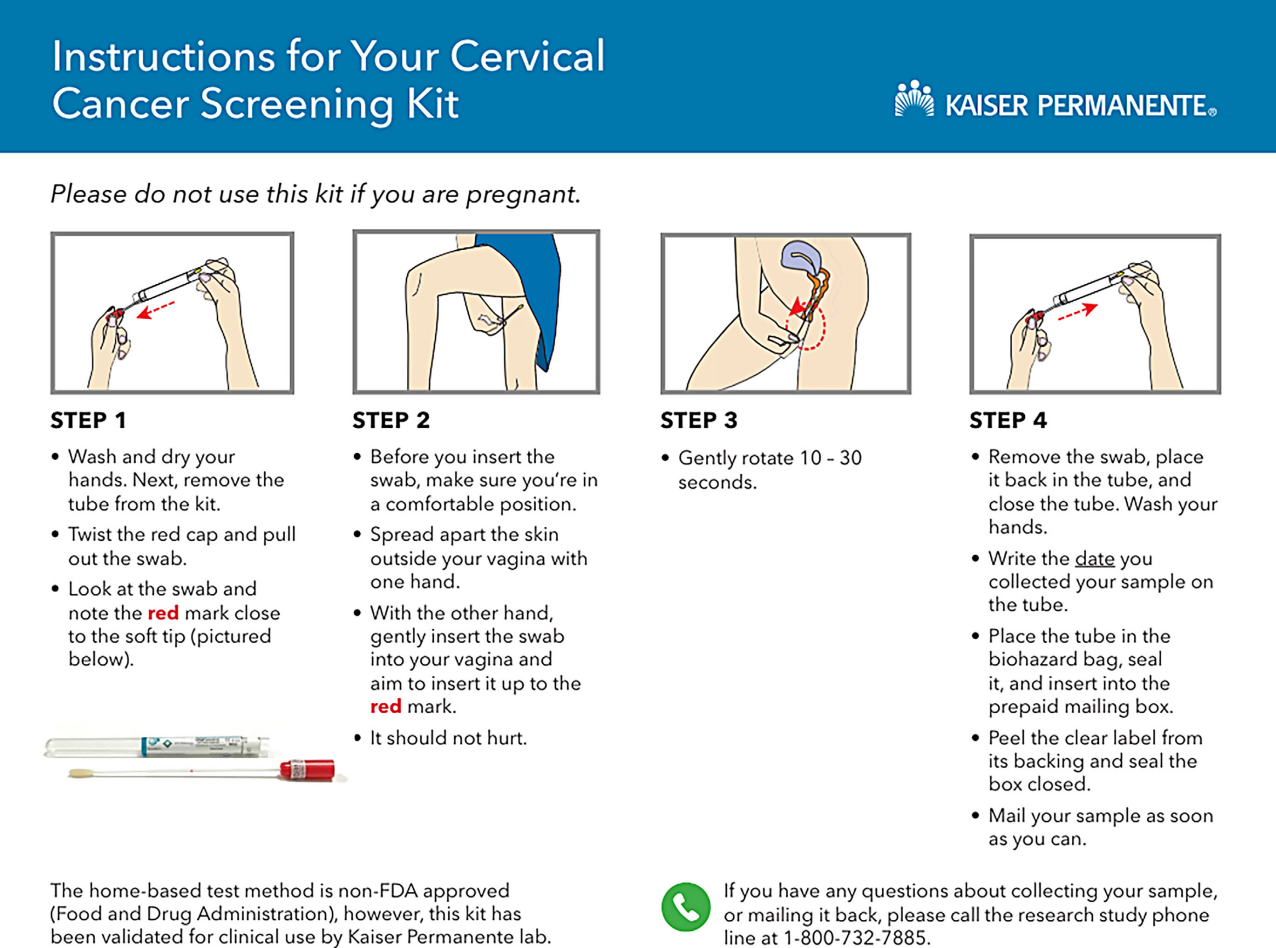
Self‐test instructions.

A prior study (the STEP trial) tested the effectiveness of at‐home HPV self‐sampling at Kaiser Permanente Washington (KPWA).^16^ Patients in the direct mailed group of STEP received a packet that included illustrated instructions (Figure 1). Here, we sought input on the STEP instructions and a sample introductory letter (Appendix) that could be used by KPNW with Black/African American, Hispanic/Spanish speaking, transgender/nonbinary, and American Indian/Alaska Native patients.

### 2.1 Recruitment

We sought to obtain a range of cultural perspectives from individuals from underserved communities who are at risk of not receiving cervical cancer screening, so we recruited among Black/African American, Hispanic (both bilingual and monolingual Spanish speaking), transgender/nonbinary and American Indian/Alaska Native populations. Our goal was to complete 20 in‐depth interviews. We completed 23, a number we have found to be sufficient in obtaining robustness of findings and saturation for content analysis, based on both our experience and qualitative literature.^17^, ^18^ The KP Interregional IRB (KPiIRB) determined this quality improvement project does not meet the regulatory definition of research involving human subjects, but recruitment letters contained elements of consent, and patients' consent to recording was confirmed prior to the interview. Patients were informed that we may publish the results, which may include quotes from the interviews, but we will not publish their name or other identifying information.

A KPNW analyst identified eligible participants for recruitment from the electronic health record. Patients were eligible if they were between the ages of 30 and 65, current KPNW members, assigned female sex at birth and from underserved groups. Recruitment targeted at least 20 interviews; 5–7 from each group: Black/African American, transgender/nonbinary, American Indian/Alaska Native and Hispanic (aiming for five bilingual and two monolingual Spanish interviewees).

Study data were collected and managed using REDCap electronic data capture tools hosted at the Kaiser Permanente Center for Health Research. REDCap (Research Electronic Data Capture) is a secure, web‐based software platform designed to support data capture for studies. Patient information for recruitment was uploaded to REDCap and qualitative interview staff were assigned to each group based on cultural competence.

Staff (C.A.J., A.F.P., S.G.) mailed recruitment letters in Spanish or English to 200 participants (50 were randomly selected in each group), and patients were given a private study voicemail number to call if they were interested in participating. Staff checked the study voicemail daily and followed up directly to schedule interviews with interested patients. Qualitative interviewers (J.S.R., A.J.F, C.A.J., and B.W.L.) also made outreach recruitment phone calls to the randomly selected patients until recruitment goals were met for each group. Patients were offered a $25 gift card for completing the interview.

### 2.2 Data collection

Qualitative interviewers sent participants a link to an informational video (1 min, 34 s) via text message, and a PDF of the Self‐Test Instructions (Figure 1) and the informational letter (Appendix) by email prior to scheduled interviews. The instructions and letter were translated into Spanish for Spanish‐speaking participants by a Kaiser Permanente certified translator.

The study team (J.S.R., A.J.F., A.F.P.) developed a semi‐structured, open‐ended interview guide to use during the patient interviews. The guide was informed by the Learner Verification and Revision (LVR) model, which is designed for assessment and development of educational materials by assessing concepts such as “attraction” and “cultural‐linguistic acceptability.”^19^ The guide included questions about the letter and instructions, asking specifically about the appeal of the screening method (attraction), the participant's ability to understand the information (comprehension), participant confidence in being able to do the test (self‐efficacy), and if the materials were culturally appealing (cultural‐linguistic acceptability). Participants were asked for specific recommendations to the messaging and instructions. Interviews were conducted by telephone using Microsoft Teams between October and November 2023; each lasted about 30–45 min. Patient interviews conducted in Spanish (by J.S.R., a trained bilingual and bicultural interviewer) were kept in the source language for analysis to ensure an accurate representation of the patient's experience. Two masters‐level study staff with qualitative interviewing training and experience (A.J.F., J.S.R.) led the data collection and analysis process with the assistance of two additional trained qualitative study staff (B.W.L., C.A.J.).

### 2.3 Analysis

The interviews were audio‐recorded and English interviews were transcribed using Microsoft Teams. Following each interview, interviewers used a brief topical content analysis approach to summarize the interview data. When necessary, they referred to the transcript for additional details and quotes.^20^, ^21^ The data were analyzed utilizing an organizational system, which categorized patient responses from each of the LVR elements into comprehensive summary tables. Patient transcripts were summarized by A.J.F., J.S.R., B.W.L., and C.A.J., working to identify themes and explore and compare variation within each LVR element for each patient group. Quotes were included to illustrate findings within each element. Spanish language interviews were summarized and reviewed by J.S.R. to facilitate understanding (e.g., cultural variations and idiomatic phrases). Multiple reviews of individual interview summaries against the LVR concepts led to identifying the emergent themes from the interviews and to a consolidated thematic summary report for each group. These summary reports formed the basis of the findings reported in this manuscript. Each report included recommended messaging and modifications to the instructions.

## 3 RESULTS

Interviews were conducted with 23 patients (Table 1). We mailed 200 recruitment letters; 3 patients called to opt‐in and 68 patients were called at least once by phone. Of those called, 6 declined to participate in the interview, and 42 passively refused by not responding to outreach attempts. Our sample included five Black/African American patients (21.7% of the sample), five Hispanic Spanish‐speaking patients (21.7%), two Hispanic English‐speaking patients (9.0%), six transgender/nonbinary patients (26.0%), and five American Indian patients (21.7%); no participants were members of more than one demographic group. The average participant age was 45 years and 73.9% of participants were up to date on cervical cancer screening.

**TABLE 1:** Patient characteristics.

	Black/African American (AA)	Hispanic (Spanish Speaking) (SP)	Transgender/Nonbinary (TG)	American Indian (AI)	Total
Interviews completed	5	7	6	5	23
Age range	32–58	39–59	31–56	30–53	30–59
Average age	42	46	43	45	45
Language (% English)	100.0	28.6	100.0	100.0	78.3
Prior screening (% up to date)	100.0	28.5	83.0	100.0	73.9

### 3.1 Initial reactions to the self‐test

Prior to the interview, interviewers ensured that participants had reviewed the video, letter, and instruction sheet. Participants were asked about their perceptions regarding the test as a screening option, their feedback on the letter and instruction sheet, and their preference for completing the test at home or in the clinic. Across all groups, participants expressed a consensus that the test would be straightforward and convenient (Table 2.).

Self‐test option is convenient, no co‐pay, no undressing, no travel, no waiting around in medical office … It's gonna be a time saver for younger people because a lot of folks don't wanna do all that. I think that they [younger people] are probably more open. AA4

It's a good idea because a lot of times it's hard to get an appointment and we are always working and it's difficult to go into the clinic. SP4

I would do this test … Perfectly fine and comfortable administering. AI2

**TABLE 2:** Reactions to the HPV self‐test.

	Initial reactions to the test and letter
African American/Black	‐ Convenient ‐ Concern about doing it correctly ‐ Looks easy, painless ‐ Mixed, some prefer to complete in office, some at home ‐ Scary when the word “cancer” is used ‐ Would open letter from KP ‐ Want to complete
Spanish speaking/Hispanic	‐ Prefer this, would do it immediately (clinic and home options, mail and clinic return) ‐ Like privacy, self‐administration ‐ Convenient (busy schedules) ‐ Prefer an alert from KP first ‐ Wonder about accuracy compared to PAP ‐ Materials are easy to follow
Transgender/nonbinary	‐ Straightforward ‐ Enthusiastic/glad about this option ‐ Self test avoids pain/discomfort/invasiveness of traditional screening ‐ Avoids misgendering and triggering dysphoria from clinic ‐ Frees up providers to do more ‐ Concern about automatic opt‐in to receiving test ‐ Would feel suspicious/concerned if received test without warning ‐ Complete at home (*n* = 3) or clinic (*n* = 1), but would want warning if in clinic
American Indian	‐ Straightforward, well branded from KP ‐ Seeing OB/GYN is scary and anxiety provoking, invasive, awkward, uncomfortable ‐ Convenient ‐ Home test/clinic test is preferable ‐ More info that this is not scraping cervix (less painful)/not same as PAP

Abbreviation: HPV, human papillomavirus.

All four groups included participants who indicated that they would be willing to perform the test at home and in the clinic.

Sometimes there are time limitations and sometimes you don't have time to go to the clinic so just having that option is really nice especially for someone who maybe has to rely on transportation so being able to do certain things at home and take those tests is super helpful. And the comfort level are the parts that I like and spoke to me. SP7

I will be comfortable doing it at my doctor's office and leaving the sample there.

I just feel like my choice was influenced by, I guess, my worries of contaminating the sample. AA5

Doing it in the clinic the first time might be better [than doing it at home] because then you can have some support if you need it. TG4

All six transgender/nonbinary patients noted preference for completing at home, but four also reported willingness also to complete in the clinic.

I would feel good [completing the self‐test]. For me as a trans guy with a, you know, vagina and a uterus … it's just awkward to go in to do gynecological things. So, like, anything that can save me from doing that I will do so happily … Being able to do this at home seems really positive. TG4

Transgender/nonbinary (*n* = 3) and American Indian (*n* = 5) populations mentioned that the test seemed straightforward and easy to understand. “It seemed really straightforward—I was surprised … Going in for like a pap is uncomfortable and painful, so I was surprised that you could do it at home to begin with and that it's not supposed to be painful. And that you don't have to go get a pap if everything looks normal.”—TG5.

Patients from the American Indian and transgender/nonbinary populations stated that they liked the test because it avoids the pain, discomfort, and invasiveness of traditional screening.

I was impressed that you guys were on top … of even trans‐guys still have vaginas. I thought it was great that it was going to be a do‐at‐home thing. Especially for trans‐guys. Going into a gynecologist's office can be a little triggering, so having something that saves you from that is great. TG1

[I would] do anything to not have to get into stirrups … [the pap] exam feels humiliating. Anything that would be an alternative with more dignity saves time and avoids a doctor appointment is a good thing. AI2

Patients from the American Indian, transgender/nonbinary, and Hispanic populations identified that seeing a provider for a pap exam can be anxiety provoking.

I know I need [to get screened] but just thinking about doing [a pap] makes me feel uncomfortable … Even knowing it's a female doctor doing the exam doesn't make me feel comfortable. SP5

Patients from the Hispanic, transgender/nonbinary and the Black populations identified preferring an alert or letter from KP before they receive the test.

I just wanted to make sure that we're getting the message out to all of all the customers [KP members], especially the people of color. I think a lot of us are not keeping our annual exams, and a lot of us are the ones that are being mostly affected that it's not getting to our people. AA4

Personally, I would prefer to opt in just so that I'm not confused by why someone is sending me something, like if I were sent this without any context, I probably wouldn't do it. TG3

### 3.2 Reactions to the materials

Patients were then asked about what they liked or disliked regarding the letter and instruction sheet, encompassing aspects such as their visual appeal, length, amount of text, color usage, inclusion of images, Spanish translation, and identification of any unhelpful or unnecessary information. Overall, all patients expressed satisfaction, perceiving both the letter and instruction sheet as visually appealing and easy to understand (Tables 3 and 4). They also reported that there was sufficient information which instilled confidence in their ability to complete the test effectively. They appreciated that the instruction sheet and letter stressed the importance of screening. Most participants across all groups felt the content was sufficient.

**TABLE 3:** Reactions to the letter.

	Attraction	Comprehension	Self‐efficacy
African American/Black	‐ Liked the reminder ‐ Loved additional resources ‐ Professional, liked the colors and font ‐ Just the right amount of information ‐ One person said too short; but majority said just right ‐ Picture grabs your attention, could be bigger, one person put off by picture	‐ Easy to understand	‐ Enough information to do the test, importance of screening, and for members due
Spanish speaking/Hispanic	‐ Complete, clear, easy to understand (all 7) ‐ Gives right information; likes FREE ‐ Likes video link and phone number ‐ Add about quality of test, and it works, and provides accurate results ‐ Likes translation	‐ Not confusing ‐ Quick blurb about next steps ‐ Add more about screening recommendations	‐ Yes, enough information and education to understand
Transgender/nonbinary	‐ Do not like “biological females” (*n* = 4) vs. likes phrase (*n* = 2) ‐ Likes illustration (*n* = 3); vs. illustration might make uncomfortable (*n* = 2) ‐ Likes bullet points, easy to read through compared to wall of text ‐ Would pick up letter to read it ‐ Likes that letter does not use Mr. or Mrs.—feels more inclusive ‐ Likes American Cancer Society recommendation, Kaiser branding, trustworthy	‐ Easy to understand/good use of plain language ‐ Readers might be unclear if self‐test is for HPV, cancer, or both, and what cervical cancer is; more info on what colposcopy is ‐ Unclear on how self‐test would happen in the clinic/where would ppt go? ‐ Consider more labels on picture, e.g., uterus, vagina, urethra	‐ Enough information to understand why they would be receiving the test ‐ Likes that letter does not assume everyone has knowledge of why they would be getting the letter ‐ ppt noted not flexible enough to see where the red line is on the swab—having a different texture at that point would help ppt know they had inserted the swab far enough
American Indian	‐ Good overview ‐ Images helped; image looks terrifying—remove from letter or different image	‐ Easy to understand, concise ‐ Concern that kit could be tampered with if sending back ‐ Possible concern about not doing correctly	‐ Enough information ‐ More information about why do test ‐ There needs to be something from KP telling people that there is this new self‐test that they will be getting, or even asking if patients want to get it

Abbreviation: HPV, human papillomavirus.

**TABLE 4:** Reactions to the instructions.

	Attraction	Comprehension	Self‐efficacy	Culturally acceptable
African American/Black	‐ Picture small (1) ‐ Attracted to picture, or describes steps (3)	‐ Liked colors and pictures of each step ‐ Intimidating until watched the video ‐ Straightforward, easy to follow, made sense	‐ Would do the test ‐ Could not follow other instructions, but can these	‐ Not offensive
Spanish speaking/Hispanic	‐ Images easy to follow	‐ Easy to understand ‐ Increase font size ‐ More information about how to prepare (shower, sex, time of day, period, laying down)? ‐ Exclusions?	‐ Enough information to do the test ‐ Could send more info about HPV and cervical cancer	‐ Translation is done well ‐ “Home test” in addition to “self‐test” ‐ “Cotonete”—next to “hisopo” ‐ Images are helpful for reading barriers
Transgender/nonbinary	‐ Would want to pick up and read it ‐ Instructions seem clear, approachable ‐ Change skin color on illustrations ‐ Be aware of traditional representation of a woman (in a dress) for the instructions ‐ Likes Illustrations	‐ Easy to understand/good use of plain language ‐ No unnecessary information to be removed ‐ Consider more labels on pictures, e.g., uterus, vagina, urethra ‐ Make time frame for sending in test clear ‐ Make red mark on test stick clearer/more obvious ‐ Highlight more that the test should not hurt ‐ Consider changing font colors to be more friendly to color‐blind patients	‐ Instructions would motivate to complete self‐test ‐ Feels pretty confident after reading instructions ‐ Including information from FAQs would make ppt more confident	‐ Use of phrase “biological females” is uncomfortable ‐ Inclusion of picture in letter might make reader uncomfortable ‐ Use of phrase “excesses force” seems out of place, discomforting ‐ Aware that talking about sex organs might make some readers uncomfortable, but sees that as unavoidable/nothing overly graphic
American Indian	‐ Looks clinical ‐ Use more color ‐ Well branded ‐ Images make less intimidating ‐ Likes QR code	‐ Easy to understand ‐ Nothing offensive ‐ Include time frame for when to send back	‐ No additional information necessary ‐ Confident in doing test ‐ Red text can be problematic for color blind	‐ Will improve access for community ‐ Consider brown skinned person ‐ Consider adding “no shame” in doing the test yourself ‐ Good option for underserved population

Abbreviation: HPV, human papillomavirus.

Black/African American patients liked the content, pictures, and thought the materials contained just the right amount of information.

As a matter of fact, I think the picture gives it more, makes it more appealing to want to read versus just some letter because a lot of people will just throw it away. AA2

All Hispanic patients said that the materials were easy to understand (*n* = 7), and that they gave the right information. They thought the materials were not confusing, but could be enhanced by adding more about the next steps and screening options.

The images are really helpful because it shows you how to prepare yourself and what to do. SP1

Spanish‐speaking patients were very happy to see instructions translated into Spanish, with clear illustrations to address language and literacy barriers.

My mom is not a strong reader, she comes from Colombia and her reading is very little, so the pictures really help people that aren't strong readers. SP7

I don't speak any English and I was able to understand everything. SP2

Transgender/nonbinary patients found the materials easy to understand and liked the branding and information in the letter. However some transgender/nonbinary patients did raise concerns. Specifically, four patients did not like the terminology “biological females,” while the other two patients liked the phrase. Similarly, two patients found the illustration troublesome and thought it might make transgender/nonbinary patients uncomfortable, but three others liked the illustration.

One thing in the letter … that got to me–is ‘biological females.’ Which technically, yeah. But I kind of wonder if, like, ‘people who have cervixes’ [could be better]. Because there are also, biological females who have had hysterectomies that no longer have cervixes. And so presumably are also exempt from this testing. So replacing that phrase of ‘biological females’ with, like, ‘patients with cervixes’ might be a little more precise … It would definitely land differently. TG1

I also appreciated that the letter has the recommendation for biological females–wording I appreciate. I'm queer nonbinary so that language is more comfortable for me. And I think probably for a lot of people. TG5

American Indian patients noted that the materials were concise with enough information and a good overview.

Right to the point and said what needed to be said. AI3

Members of all groups regarded the materials favorably, but all groups had suggestions for making the materials more culturally acceptable (Table 5). Specifically, Black patients suggested clarifying the information about when to return the test, how tests would be resulted, and to avoid using the term “cancer.” Spanish‐speaking Hispanic patients had specific language suggestions, like using “prueba casera (home test)” and “cotonete (cotton swab)” to describe the test. American Indian patients stressed the importance of highlighting how this test allows for convenience and privacy.

Also for people who don't [have easy access to medical care] and are a little more uncomfortable being advocates for their own health. This is something that's private and it's easy. AI3

If it mentioned that this is something that you're wanting to do to provide women with, like women these days can use all the more privacy and respect. AI2

**TABLE 5:** Recommended changes to the materials.

	Letter	Kit instructions	Identified barriers to screening
African American/Black	‐ How long to get test results ‐ Do not use word cancer ‐ Picture can be off putting	‐ Include video link ‐ Clarify to mail back w/in a few days	‐ Lack of time ‐ Lack of health insurance
Spanish speaking/Hispanic	‐ Stress prevention ‐ Clarify comparison to PAP ‐ Slightly larger text ‐ Add visuals to letter ‐ Add age and screening intervals ‐ Phone number for questions about cervical cancer ‐ Clarify next steps ‐ Clarify more about the cost of the test	‐ Increase font size ‐ More information about how to prepare (Shower, sex, time of day, period…) ‐ Clear return date ‐ “Autoprueba (self‐test) vs. Prueba casera (home test)”. ‐ Add “home test” in parenthesis next to “self‐test” for older generations. ‐ Could also add “cotonete” (cotton swab) next to “hisopo”	‐ Nervous about doing pap
Transgender/nonbinary	‐ Change language to “people with cervix's” ‐ Be sure not to use Mr./Mrs. ‐ Rather than naming specifically what follow‐up tests might be, change language to be more broad ‐ Consider more labels on pictures, e.g., uterus, vagina, urethra ‐ Clarify what HPV stands for, information on cervical cancer; clarify if test is for HPV, cancer, or both ‐ Consider changing font colors to be more friendly to color‐blind patients	‐ More info on red line on the test, not able to “see” when inserting ‐ Consider FAQ's document ‐ Change skin color on illustration, change purple color of uterus, change color dress in illustrations ‐ Clear phone number; include KP phone number with list of warnings and time frame for sending in test ‐ Highlight that the test should not hurt ‐ Clarify where QR code will take reader (video) ‐ Clarification/illustration to clarify some people can get confused about terms for anatomy; more picture labels	‐ Experiences gender dysphoria/vulnerability ‐ General discomfort ‐ Attending to other chronic health conditions ‐ Cost/insurance ‐ Inconvenience, not clear on screening timelines ‐ No primary care provider (PCP) ‐ PCP lack of awareness not straight women (i.e., pregnancy questions)
American Indian	‐ Consider changing self‐test to be more specific, softer intro, stress convenience and privacy, and that you are due ‐ Spell out HPV, emphasize detection ‐ Clarify mailing/in‐clinic, add information link, why do at home ‐ Clarify steps for follow‐up, add time frame ‐ Change “we want you” to “it is beneficial” ‐ Remove “family”—not all have it ‐ Red text difficult to see	‐ Highlight “it should not hurt” ‐ Clarify what test is made of ‐ Clarify process of getting kit (mailed vs. in clinic) ‐ Clarify prep steps (does vagina need to be cleaned)	‐ Time off to go to a medical appointment ‐ Notifications from physician that due, and testing options

Abbreviation: HPV, human papillomavirus.

Transgender/nonbinary patients also made suggestions to improve the content and wording, including highlighting that the test should not hurt.

You can tell that it's a feminine‐ish person, but it's not like an overly womanly‐looking person either. It's sort of–it's more just a little closer to the kind of a neutral body thing … that part felt really inclusive. TG4

This may be a problem more for trans guys than anyone else … unless we're really diligent about putting on estrogen cream … it can get painful down there … which is why I would put: ‘can I put a little lube on there to ease the process?’ TG1

Overall, improvements were identified to make both the instructions and the letter more culturally appropriate for each group.

## 4 DISCUSSION

In interviews, patients from Black/African American, American Indian, transgender/nonbinary and Hispanic and Spanish‐speaking populations reported finding the HPV self‐test, instructions, and introductory letter acceptable in terms of attraction, comprehension, self‐efficacy and cultural appeal. However, members of all groups suggested improvements to make materials more culturally appealing, or tailored for diverse cultural groups. Considering these components and tailoring materials for the needs of specific groups may be key to increasing response rates and reducing disparities in cervical cancer screening and outcomes.

In designing HPV self‐sampling interventions and outreach, healthcare organizations must recognize that individual patients may have different concerns or needs based on culture, identity, and past experiences with the healthcare system. Historically underserved groups have experienced healthcare disparities, and organizations must be intentional about rolling out new tools in ways that counteract, rather than perpetuate, existing inequities. This can be achieved by making specific modifications to outreach materials to tailor them to historically disadvantaged groups.^22^, ^23^

For example, in regards to transgender/nonbinary patients, studies have identified predominant issues regarding ongoing patient misgendering, and a lack of gender‐neutral hospital and health care environments.^24^ This can be particularly important to recognize in cervical cancer screening, as people with a cervix who are transgender/nonbinary are at particular risk of misgendering which can exacerbate gender dysphoria.^25^ In our interviews, transgender/nonbinary people were excited about a screening alternative to a pelvic exam, and suggested tailored language to ensure that materials felt inclusive and non‐triggering, increasing the likelihood of follow‐through with screening.

Tailoring interventions could impact healthcare quality and facilitate addressing the causes of inadequate screening. In lung cancer, tailored approaches to engage diverse populations based on the social ecological model were shown to address disparities in screening.^26^ Patient navigators have also been able to address multilevel factors by using motivational interviewing to address cultural norms and bring cultural relevance to the importance of screening.^26^ Marginalized individuals may be more likely to engage in health utility maximizing behavior if they feel the likelihood for future discrimination is minimal.

This qualitative study highlights ways to tailor materials to specific populations, as the participants made suggestions that should be considered for each group. The Black/African American participants recommended removing the word “cancer” from materials, specifying how long patients would have to wait for test results, and clarifying that the test should be mailed back in a few days. The Spanish‐speaking participants gave translation recommendations to make messaging clearer, and asked for clarifications. The transgender/nonbinary participants suggested the materials should have specific messaging for their population, including use of the phrase “people with a cervix”. The American Indian participants suggested materials should be more descriptive of the test itself, be more colorful, and stress the convenience and privacy of this test. These suggestions are easy to execute, and should be considered, as they may improve response rates and reduce cervical cancer disparities.

Limitations include we were only able to interview 23 patients in four population groups, and tailoring materials for other populations should be considered. These patients were all KPNW patients, all of whom have insurance. Future areas of qualitative inquiry should interview more patients from diverse health systems across multiple geographical regions. The participants did not have the test in hand, had never completed the test, were not asked to perform the test. Soliciting feedback after tests are performed could identify additional helpful tailored modifications.

## AUTHOR CONTRIBUTIONS

**Amanda F. Petrik:** Conceptualization (lead); funding acquisition (lead); investigation (lead); methodology (lead); supervision (lead); visualization (lead); writing – original draft (lead). **Jennifer S. Rivelli:** Data curation (equal); formal analysis (equal); writing – review and editing (equal). **Alison J. Firemark:** Data curation (equal); formal analysis (equal); writing – review and editing (equal). **Cheryl A. Johnson:** Data curation (equal); formal analysis (equal); writing – review and editing (equal). **Blake W. Locher:** Data curation (equal); formal analysis (equal); writing – review and editing (equal). **Sara Gille:** Project administration (equal); writing – review and editing (equal). **Matthew J. Najarian:** Writing – original draft (supporting); writing – review and editing (equal). **Alexandra M. Varga:** Data curation (equal). **Jennifer L. Schneider:** Formal analysis (equal); methodology (equal); writing – original draft (equal). **Beverly Green:** Writing – review and editing (equal). **Rachel L. Winer:** Writing – review and editing (equal).

## FUNDING INFORMATION

Resources for this study were made possible by the Learning Health System Program at the Kaiser Permanente Center for Health Research.

## CONFLICT OF INTEREST STATEMENT

All other authors declare no conflicts of interest.

## ETHICS STATEMENT

Ethical approval was sought from an Institutional Review Board (IRB) prior to commencing this study. The KP Interregional IRB (KPiIRB) determined this quality improvement project does not meet the regulatory definition of research involving human subjects, but recruitment letters contained elements of consent, and patients' consent to recording was confirmed prior to the interview.

## Data Availability

Aggregated qualitative data is available upon request to the corresponding author.
